# Keeping their own and integrating the other: medicinal plant use among Ormurs and Pathans in South Waziristan, Pakistan

**DOI:** 10.1186/s13002-023-00634-z

**Published:** 2023-12-17

**Authors:** Muhammad Abdul Aziz, Musheerul Hassan, Aman Ullah, Zahid Ullah, Renata Sõukand, Andrea Pieroni

**Affiliations:** 1 Department of Environmental Sciences, Informatics and Statistics, Ca’Foscari University of Venice, Via Torino 155, 30172 Venice, Italy; 2 University of Gastronomic Sciences, Piazza Vittorio Emanuele II 9 Bra, 12042 Pollenzo, Italy; 3 Department of Ethnobotany, Institute of Botany, Ilia State University, 0105 Tbilisi, Georgia; 4 Department of Zoology, Alpine Institute of Management and Technology, Dehradun, Uttarakhand 248007 India; 5 Faculty of Education and Social Sciences, Shaheed Zulfikar Ali Bhutto Institute of Science and Technology, Karachi, Pakistan; 6 Center for Plant Sciences and Biodiversity, University of Swat, Kanju, 19201 Pakistan; 7 Department of Medical Analysis, Tishk International University, Erbil, Kurdistan 4401 Iraq

**Keywords:** Medical ethnobotany, Pathans, Ormurs, Medicinal plants, Waziristan, Kaniguram

## Abstract

**Background:**

In multicultural societies, traditional knowledge among minorities faces several challenges. Minority groups often face difficult situations living in specific peripheral geographies and striving to retain their biocultural heritage, including medicinal plant knowledge and practices. Folk medicinal plant knowledge is a dynamic eco-cultural complex influenced by various environmental, socio-cultural, and political factors. Examining medicinal plant knowledge among minorities has been an increasingly popular topic in cross-cultural ethnobiology. It also helps understand the dynamics of local/traditional ecological knowledge (LEK/TEK) change within a given community. The current study was designed to investigate the status of medicinal plant knowledge among two linguistic groups, i.e. Ormurs and Pathans, living in a remote valley of West Pakistan.

**Methods:**

We recruited 70 male study participants from the studied groups for semi-structured interviews to record the medicinal plant use of their communities. Data were compared among the two studied communities using the stacked charts employing the presence or absence of data with Past 4.03 and Venn diagrams. Use reports (URs) were counted for each recorded taxon.

**Results and Discussion:**

A total of seventy-four medicinal plants were quoted as used as ethnomedicines by the researched communities. Most of the reported plants were used to treat digestive and liver problems. The cross-cultural comparison revealed a considerable homogeneity of medicinal plant knowledge (the two groups commonly used more than seventy plants); however, comparing uses recorded for the widely utilised medicinal plants showed numerous idiosyncratic uses among Ormurs but very few among Pathans. Ormurs reported a higher number of cultivated, wild, and imported plant uses than did Pathans. These results indicate that, compared to Pathans, the Ormur linguistic minority retain more folk medicinal plant knowledge, which may be explained by the fact that they have incorporated different folk remedies: their “own knowledge” plus that of Pathans, with whom they have lived together for centuries. Moreover, the local plant nomenclature among Ormurs was highly affected by the plant nomenclature of Pathans.

**Conclusion:**

The current study revealed that living together for a few centuries has not implied sharing plant knowledge (as the Pathans do not seem to have learnt from the Ormurs) or, in other words, that plant knowledge exchanges have been unidirectional. The findings show that the Pashto dominant culture may have possibly put pressure on the minority groups and affected local plant-centred cultural practices, as we see in the case of local plant nomenclature hybridisation among Omuri speakers. Hence, it is imperative to employ diverse educational strategies to revitalise the decline of medicinal plant knowledge in the studied communities, especially among Ormurs, who need more attention as they face more challenges than the other group. Locally based strategies should be devised to restore the fading connection with nature, which will be advantageous for revitalising plant knowledge.

## Background

In multicultural environments, minority groups adopt the dominant way of thinking, practising, and behaving. Being part of the system, cultural knowledge, including local plant knowledge, is also impacted and subjected to change in multicultural societies [1]. A few researches have shown that sharing local plant knowledge among different cultural groups is often a phenomenon of cultural assimilation and standardisation towards the dominant cultures (see, for instance, [2–5]). Cultural assimilation is triggered by historical, political, and religious pressures, resonating in the daily practices of local communities. As a result, the attached local plant knowledge and its transmission are also greatly affected.

Field ethnobotanical studies across Pakistani Hindukush have revealed that local ecological knowledge related to both medicinal and food plants is highly threatened among the local communities, including minority groups [3–6]. Moreover, in multicultural environments, language vitality among minorities has been severely affected by sociolinguistic adaptation (see, for instance, [4, 6]). It has been claimed that the erosion of the vernacular names of plants among minorities may indicate the decline or homogenisation of traditional or local knowledge within each minority group. For instance, a study conducted in Kaniguram among Ormurs and Pathans revealed considerable homogeneity in using wild food plants (WFPs) [6]. In addition, our research has indicated that Ormurs have adopted the local Pathan names of many WFPs, as Pashto is spoken as the *lingua franca* in the study area. Ormurs are a cultural diaspora from the Middle East that arrived in the area during the eleventh century and represent a tiny minority group living in the small valley of Kaniguram in South Waziristan (see [7, 8]. Their language is highly threatened [9]. Given the [6] results, we have conducted a cross-cultural ethnomedicinal study among Ormurs and Pathans to identify the patterns of knowledge distribution of medicinal plants among the two communities. The food ethnobotanical study conducted among the two groups revealed that knowledge of WFPs is primarily shared among the two communities; however, the dynamics of medicinal plant knowledge transmission are not the same as for WFPs [10]. However, medicinal plant knowledge is shaped in a particular way within a given cultural group, which certain factors could influence. Therefore, it stands to reason to investigate the patterns of medicinal plant use among Ormurs and Pathans as we think that the knowledge and practices of these two groups may differ [11].

In a broader sense, cross-cultural study will help to understand how local or traditional plant knowledge evolves and is reshaped among different cultural groups [12–17]. Thus, a cross-cultural analysis might provide a reflection of the spatiotemporal changes in past plant uses, which can be used to establish and interpret the results in a historical context, as Olsson and Folke observed [18] that the specific characteristics of traditional ecological knowledge (TEK) lie in its “historical and cultural continuity of resource use”.

It is important to note that this study is the very first scientific investigation in North and Western Pakistan that takes into account sound ethnobotanical standards to study the local plant knowledge on medicinal plants to provide better scientific interpretations for future research work regarding the sharing of knowledge of medicinal plants across different cultural groups, including minorities. In the region, hundreds of studies have already been carried out in the past decades to document the therapeutic uses of local plants (see for reference: *Journal of Ethnopharmacology*, *Journal of Ethnobiology and Ethnomedicine*, *Frontiers in Pharmacology*, *Plos One*, and many other international and national journals); however, we have often observed vague scientific data interpretations made in these studies, and a few of these researches do not even follow some of the essential ethnobotanical standards recommended by ethnopharmacologists (see [19, 20]).

We have therefore conducted a cross-cultural ethnobotanical study with the hypothesis that the medicinal knowledge of Ormurs may have been impacted by their nearby Pathan neighbours, which led us to formulate an overarching research question regarding the vulnerability of the Ormuri ethnomedical system due to a possible homogenisation imposed by the dominant Pathan group. Ormurs and Pathans live close to each other and share marriages, values, cultural richness, and knowledge of daily life practices.

The specific research objectives of the study were:

1. To document the plants’ local names and medicinal uses among the two linguistic communities;
2. To compare the medicinal plant reports among the two groups.

## Materials and methods

### Study area and ethnic groups

**Fig. 1:**
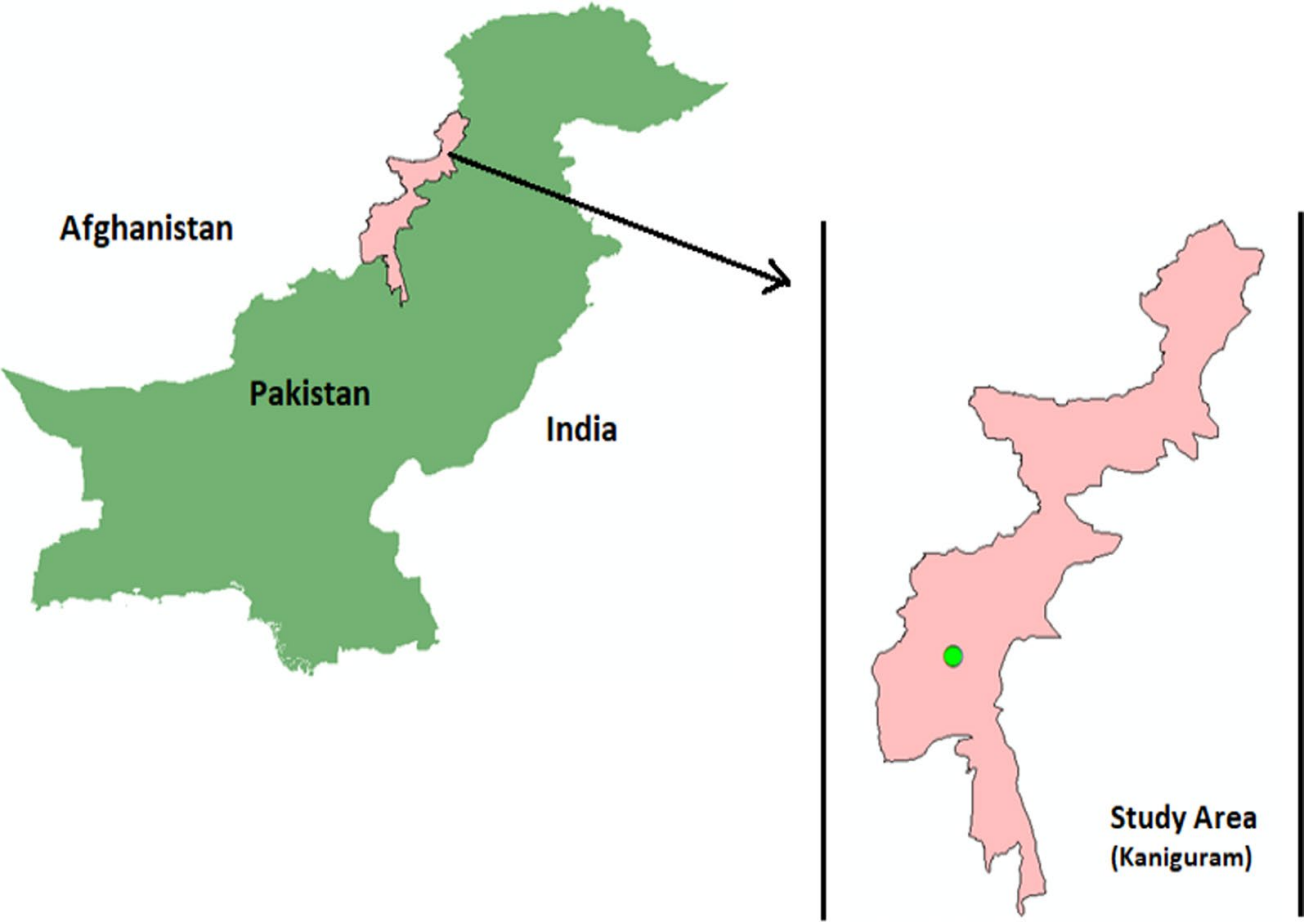
Map of the study area

The present study was conducted in Kaniguram, located along a sloping hillside in South Waziristan, Pakistan (Fig. 1). The study area is populated by two ethnic groups, i.e. the Ormur diaspora and Pathans, which have lived together for centuries. Pathans are the dominant ethnic group with their distinct cultural heritage, attributed to their specific traditions, customs, and *Pashtunwali*—the Pathans code of life. In Kaniguram, Pashto is spoken as the language of communication or the *lingua franca,* while Urdu is the primary language officially used in schools and administrative offices.

The ancient Ormur diaspora is a distinct linguistic minority that originated in the Middle East in the eleventh century. Khattak [21] reported 10,000 speakers of the Ormuri language in the study area, and now the number could be more than that. Traditions relate that Ormurs may be the descendants of Persians, Arabs, Kurds, or Afghans [7, 22, 23]. It has also been stated by some authors that Ormurs originated from the southern shores of the Caspian Sea in Persia and migrated to the south-eastern part of the Iranian territory relatively recently [24], while other viewpoints suggest they represent Indigenous communities that occupied lands south of the Hindukush from time immemorial. Their language, however, is the only surviving representative of a south-eastern subgroup of Iranian languages [7, 23, 25, 26]. Captain Leech [8] stated that the Ormur were included in the general term *Parsiwan* or *Tajak* and could have arrived from Yemen. Sultan Mahmud of Ghazni might have brought them, as they were part of his army when he invaded India in the eleventh century. The Ormur people were highly instrumental in removing the temple’s gates of Somnath [7, 8]. Later, after conquering the temple, the army returned to Afghanistan. While on their way, some of the Ormur soldiers settled in Kaniguram as they found the valley a suitable place to live. For further details, see our article [6].

### Post-conflict social change

The “war on terror” in Pakistan, specifically in the Waziristan region, has significantly impacted the local population, including the people’s traditional plant knowledge. The war started in 2004, and the security situation remains highly fragile. The ongoing conflict has resulted in the large-scale displacement of people, the destruction of infrastructure, and the disruption of livelihood activities. Because of the displacement, many people have lost access to their traditional lands and resources, which has resulted in a loss of knowledge about local plants and their uses. The destruction of infrastructure has also had an impact, as many traditional medicinal practitioners, who were the custodians of this knowledge, were forced to flee their homes, and their knowledge was not passed on to the next generation.

Additionally, the militarisation of the area has limited access to the land, which makes it difficult for people to continue their traditional livelihood activities such as agriculture, pastoralism, hunting, and gathering, which are closely linked to their traditional plant knowledge. One crucial aspect is the linguistic erosion among Ormurs and the adoption of the Pashto language as the *lingua franca*. The large-scale outmigration has impacted the language as well.

### Vegetation and environment

Kaniguram, located in the upper part of South Waziristan, is a Valley surrounded by hills and rugged mountains. It is a town at 32° 31′ 7ʹʹ N and 69° 47′ 11ʹʹ E. The climate is Humid subtropical. In winter, the valley is covered with snow. The winter is extremely severe, with the coldest months from December to February. In summer, the temperature is moderate. The average annual rainfall is 6 inches, while the summer is relatively hot in plain areas. Kaniguram is located at a high elevation in South Waziristan, where vegetation cover is mainly composed of *Quercus* spp., and at even higher elevations, *Pinus gerardiana* Wall. ex D. Don, *Pinus wallichiana* A. B. Jacks., *Cedrus deodara* (Roxb. ex D. Don) G.Don, and *Abies pindrow* Royle are the common botanical taxa [27].

### Field survey

Ethnomedicinal fieldwork was carried out by the first and the second author in 2021 (October to December) and 2022 (July to August). Data were gathered through semi-structured interviews with elderly community members (aged forty-five to ninety years) among the two linguistic groups. The study participants chosen for the survey have a long-lasting relationship with the natural environment and the local flora and were recognised as experienced in local medicinal plant knowledge. Male field researchers co-authoring this article recruited male study participants in the interviews since female informants could not be approached due to cultural/religious issues. Moreover, it is also important to note that our respondents were not traditional healers or doctors but laypeople, i.e. experts in local plant knowledge who gained it orally from their elders. In the particular social and cultural context of the study area, not even women researchers would be allowed to freely approach and interview female study participants in the study area. We strictly followed the Code of Ethics of the International Society of Ethnobiology [28] while conducting the interviews. Prior oral consent was obtained from each of the participants before each interview. Seventy male participants were selected for the study, including 35 from each linguistic group. The interviews were conducted in the Pashto language, which is spoken as the *lingua franca* in the study area. To each participant, we explained the topic and objectives of the study.

We adopted a mixed approach for selecting the informants. The survey started with participants selected through random sampling, and then, once we became familiar with the study area, we adopted the snowball technique. The duration of the interviews varied, i.e. in some cases, it ended after 20 min, while in others, it lasted for hours. Only participants with long experience with nature and who remained in the study area for decades were chosen. Interviews were conducted in public gathering places, local shops, and fields, mainly after prayer near mosques where both linguistic groups gather and interact. Some people were also interviewed while working in fields. The information collected from the interviewees focused on the local names of medicinal taxa, parts used, diseases treated, and modes of preparation and application. Free listing was used to obtain a thorough knowledge of the therapeutic uses of the quoted plants. Initial free listing was attempted, but it was usually short and rarely succeeded. After that, plant remedies used for different emic disease categories were asked about, following a mind-mapping system, starting with ailments of the head (headache, cold, ear and eye diseases, sore throat, etc.) and internal organ diseases (stomach, heart, lungs, kidneys, etc.), followed by systemic disorders (joint diseases, diabetes, allergies, cancer, immune system disorders), skin-related diseases and injuries (cuts, wounds, furuncles, rush), culture-bound diseases (evil eye, nightmares, etc.), and lastly any other treated illnesses not yet named. If some of the plants included in free listings were not mentioned, the respondents’ attention was also guided to those. We also asked interviewees to provide a date for the first and last use of the plant as precisely as possible to identify when it was used. We also took some photographs and received consent to publish them if deemed necessary in the publication process.

### Identification of plants and botanical nomenclature

The recorded botanical taxa were identified in the field by the fourth author, and the specimens were assigned accession code numbers and then deposited at the Department of Botany, University of Swat, Pakistan. The botanical nomenclature was verified using the World Flora Online database [29].

### Data analysis

Data were arranged in MS Excel, and use reports were counted for each quoted taxa. Associations between plant species and the diseases treated were evaluated via stacked charts, employing the presence or absence of data using Past 4.03 across the two selected groups. The collected botanical data were arranged into two binary trends for each community, and the data were compared through Venn diagrams. Bioinformatics and Evolutionary Genomics software was used for cross-cultural comparisons between ethnic groups (https://bioinformatics.psb.ugent.be/webtools/Venn/). The International Classification of Primary Care, 2nd Edition (ICPC-2, updated March 2003), was used to categorise all diseases and treated ailments. Emic disease names were correlated with this classification system’s medicinal categories (hereafter, etic disease categories).

## Results and discussion

### Medicinal plant use and quantitative analysis

**Fig. 2:**
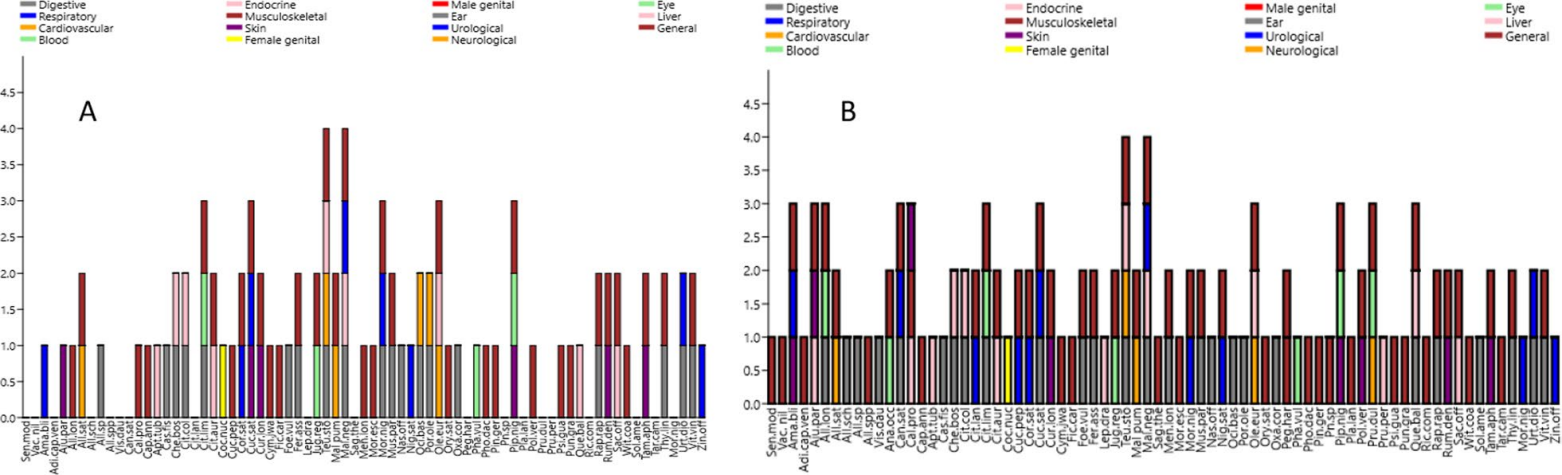
Stacked chart showing plant species used to treat various diseases among Ormurs (**a**) and Pathans (**b**) in the study area; the abbreviations refer to the first three letters of both the first and second part of the Latin binomials

We recorded a total of seventy-four medicinal plants that were quoted by the two ethnic groups in the study area. A complete inventory of the listed plant taxa is provided in Table 1. The plants were mentioned for treating various diseases, which we grouped into fifteen etic categories (Fig. 2). The dominant plant part used was fruit for both of the researched groups. The reported species belonged to thirty-nine botanical families, the most prevalent of which were Amaryllidaceae (5), Lamiaceae (5), Cucurbitaceae (4), Apiaceae (4), and Fabaceae (4); 3 or fewer plant taxa represented the remaining families.

**Table 1:** Ethnomedicinal uses of plants quoted by the two studied communities

Botanical name (Family) Voucher number	Recorded local name	Origin of the taxa	Ethnic Communities							
			Ormurs (O)				Pathans (P)			
Plants										
			Part used	Preparation	Use	Use reports	Part used	Preparation	Use	Use reports
*Adiantum capillus-veneris* L. (Pteridaceae) SWAT004625	Ashupere Sunrye/De bibiaadeSunrye^O^	Locally grown; wild	Aerial parts	Eaten with bread	Abnormal menstruation	4	–	–	–	–
					Join pain					
*Ajuga parviflora* Benth. (Lamiaceae) SWAT004626	Syed butai^O, P^	Locally grown; wild	Leaves	Fresh leaves are eaten with water	Skin rashes; infertility; diabetes	5	Leaves	Fresh leaves are eaten with water	Skin rashes	2
				Paste	Pus					
*Allium longifolium* (Kunt) Spreng. (Amaryllidaceae) SWAT004627	Khukh^O^ Yov-ree^P^	Locally grown; wild	Bulbs	Bulbs are consumed as a snack	Digestion; blood thinner; abdominal gas and pain	1	Bulbs	Bulbs are consumed as a salad	Weakness	1
*Allium sativum* L. (Amaryllidaceae) SWAT004628	Ozzha^O^ Yaza^P^	Bought	Seeds	Seeds are cooked with vegetables	Obesity	14	Seeds	Seeds are cooked with vegetables	Obesity	12
				Seeds are made into tea	Abdominal pain			Seeds are made into tea	Abdominal gas	
				Juice is made from the seeds	Heart problems			Juice is made from the seeds	Heart problems	
*Allium schoenoprasum* L. (Amaryllidaceae) SWAT004629	Gandana^O^	Bought	Aerial parts	Eaten as a salad	Digestive problems	1	–	–	–	–
*Allium* spp. (Amaryllidaceae) SWAT004630	Sareesh^O^ Shabye^P^	Locally grown; wild	Bulbs	Eaten as a snack	Digestive problems	2		Consumed as a salad	Digestion problems	2
*Allium* spp. (Amaryllidaceae) SWAT004631	Payaz^O^	Locally grown; wild	Bulbs	Eaten as a snack	Sexual tonic	1	–	–	–	–
*Amaranthus blitum* L. (Amaranthaceae) SWAT004632	Sakaak^O^ Ranzaka^P^	Locally grown; wild	Seeds	Seeds are consumed raw, eaten with water	Stop urine during the night (in children); constipation; skin dryness	9	Seeds	Seeds are cooked with vegetables	Stop urine during the night (in children)	4
*Anacardium* *occidentale* L. (Anacardiaceae) SWAT004634	Kajo/Badyan^O^	Bought	Fruit	Fruit is dried and consumed	Improve eyesight; immune booster; improve sexual desire; brain tonic	1	–	–	–	–
*Apteranthes tuberculata* (N.E.Br.) Meve & Liede (Apocynaceae) SWAT004638	Pamanai^O, P^ Pamonai^O^	Bought	Leaves	Fresh leaves are consumed as a salad	Diabetes	10	Leaves	Fresh leaves are consumed as salad	Diabetes	6
*Cannabis sativa* L. (Cannabaceae) SWAT004635	Bangara^O^	Locally grown; wild	Leaves	Leaves are dried and consumed	Cough	1	–		–	–
				Leaves are kept in water, and the water is then consumed	Remove sputum; respiratory tract infections; high blood pressure; digestion problems; brain tonic				–	
*Calotropis procera* (Aiton) Dryand. (Apocynaceae) SWAT004636	Spalmeka^O^ Saplme^P^	Locally grown; wild	Leaves	Milk like substance is extracted from the leaves, applied topically	Skin rashes; snake bites	1	Leaves	Leaves are made into a paste and applied topically	Joint pain	1
				Leaves are made into a paste and applied topically	Joint pain					
				eaves are consumed with water	Diabetes					
*Capsicum annuum* L. (Solanaceae) SWAT004637	Sheen Morch^O^ Sheen March^P^	Bought	Fruit	Consumed as vegetables	Fever	1	Fruit	Consumed as vegetables	Fever	1
*Cassia fistula* L. (Fabaceae) SWAT004639	Chumbarkhayan^O^ Turlargai^P^ Ghras goon^O^	Bought	Fruit	Fruit is kept in water, and the water is then consumed	Abdominal pain	6	Fruit	Fruit is kept in water, and the water is then consumed	Abdominal pain or gas	3
				Fruit is added to milk, and the milk is then consumed	Constipation			Fruit is added to milk, and the milk is then consumed	Constipation	
*Chenopodium boscianum* Moq. (Amaranthaceae) SWAT004640	SaagO Jungli saag^P^	Locally grown; wild	Leaves	Leaves are consumed as a salad or vegetables	Gastric problems; diabetes	3	Leaves	Leaves are consumed as a salad or vegetables	Acidity; diabetes	2
*Citrullus colocynthis* (L.) Schrad. (Cucurbitaceae) SWAT004641	Maraghunyee^O^ Maraghenyee^P^	Locally grown; wild	Seeds	Seeds are peeled and consumed with water	Abdominal pain; diabetes	5	Seeds	Seeds are peeled and consumed with water	Abdominal pain; diabetes	5
*Citrullus lanatus* (Thunb.) Matsum. & Nakai. (Cucurbitaceae) SWAT004642	Andwana^O^	Bought	Fruit	Fruit is consumed raw	Chest infections; kidney problems; respiratory tract infections; COVID-19	1	–	–	–	–
*Citrus limon* (L.) Osbeck (Rutaceae) SWAT004643	Neembo^O, P^	Bought	Fruit	Juice is extracted and drizzled on a salad; the extract is also used to make tea	Fever	5	Fruit	Juice is extracted and drizzled on a salad; the extract is also used to make tea	Fever; blood thinner	5
				Juice	Blood thinner; digestion problems			Juice	Digestion problems	
*Citrus* × *aurantium* L. (Rutaceae) SWAT004644	Malta^O, P^	Bought	Fruits	Fruit is eaten raw	Common cold	2	Fruit	Fruit is eaten raw	Common cold	1
			Fruit fibre or cover	Fruit cover or fibre is dried and consumed with water	Diabetes		Fruit fibre or cover	Fruit cover or fibre is dried and consumed with water	Diabetes	
*Cocos nucifera* L. (Arecaceae) SWAT004645	Kopra^O,P^ Gari^O^	Bought	Fruit	Water is obtained from the unripe fruit and consumed	Leucorrhoea	6	Fruit	Water is obtained from the unripe fruit and consumed	Leucorrhoea	4
*Cucurbita pepo* L. (Cucurbitaceae) SWAT004646	Kadi^O^	Locally grown; cultivated	Fruit	Fruit is consumed raw or cooked as vegetables	Urinary tract infections	3	–	–	–	–
*Coriandrum sativum* L. (Apiaceae) SWAT004647	Danya^O, P^	Locally grown; cultivated	Aerial parts	Aerial parts are made into tea	Chest infections; COVID-19	1	Aerial parts	Aerial parts are made into tea	Chest infections	1
				Aerial parts are made into a sauce-like substance locally called chutney	COVID-19					
*Cucumis sativus* L. (Cucurbitaceae) SWAT004648	Badring^O^ Badrang^P^	Locally grown; cultivated	Fruit	Fruit is consumed raw	Hepatitis; diuretic; digestive problems	5	Fruit	Fruit is consumed raw	Hepatitis; diuretic; digestion problems	3
				Small circular pies are used as a scrub	Cosmetics			Small circular pies are used as a scrub	Face smoothness	
*Curcuma longa* L. (Zingiberaceae) SWAT004649	Guleskhand^O^ Korkaman^P^	Bought	Rhizome	Rhizome is made into a powder and used topically	Wounds	3	Rhizome	Rhizome is made into a powder and used topically	Wounds; skin rashes	3
				The powdered rhizome is mixed with milk and consumed	Believed to be a strong antibiotic			The powdered rhizome is mixed with hot milk and consumed	Believed to be a strong antibiotic	
*Cymbopogon jwarancusa* (Jones ex Roxb.) Schult. (Poaceae) SWAT004650	Sakhosargari^O, P^	Locally grown; wild	Aerial parts	Aerial parts are dried and consumed with water	Malaria; typhoid	3	Aerial parts	Aerial parts are dried and consumed with water	Malaria; typhoid	2
*Ficus carica* L. (Moraceae) SWAT004651	Inzir^O^ Tugha^P^	Locally grown; wild	Fruit	Fruit is eaten raw	Weakness	14	Fruit	Fruit is eaten raw	Weakness	10
				Fruit is dried and consumed with milk	Piles; constipation			Fruit is dried and consumed with milk	Piles Constipation	
*Foeniculum vulgare* Mill. (Apiaceae) SWAT004652	Kalvo^O, P^	Bought	Seeds	Seeds are added to vegetables	Digestion problems	1	Seeds	Seeds are added to vegetables	Digestive problems	2
				Seeds are consumed with water	Cold fever					
*Ferula assa-foetida* L. (Apiaceae) SWAT004653	Eng^O^ Aang^P^	Bought	Resin	Applied topically on gums and teeth	Infected teeth	6	Resin	Applied topically on gums and teeth	Infected teeth	2
				Mixed with water and then taken orally	Pain; digestion problems; worm infestation					
*Juglans regia* L. (Juglandaceae) SWAT004655	Matak^O, P^ Motak^O^	Locally grown; wild	Fruit	Dried fruit is consumed with milk	Weakness; increases blood	3	Fruit	Dried fruit is consumed with milk	Weakness; increases blood	4
*Lepidium draba* L. (Brassicaceae) SWAT004654	Ghargasti^O^	Locally grown; wild	Leaves	Fresh leaves are eaten as a salad	Diabetes	1	–	–	–	–
*Mentha longifolia* (L.) L. (Lamiaceae) SWAT004659	Gwan^O^ Welanai^P^	Locally grown; wild	Leaves	Leaves are powdered and consumed with water or made into tea	Abdominal pain; colic pain; loss of appetite; gastric problems	13	Leaves	Leaves are powdered and consumed with water or made into tea	Abdominal pain; colic	13
*Morus nigra* L. (Moraceae) SWAT004661	Tuth^O^ Teeth^P^	Locally grown; wild	Fruit	Fruits are consumed raw	Respiratory tract infections; appetiser	2	Fruit	Fruits are consumed raw	Urinary tract infections; Digestion appetiser	3
*Malus pumila* Mill. (Rosaceae) SWAT004657	Meleez^O^ Manra^P^	Locally grown; cultivated	Fruit	Raw fruit is consumed	Tonic; heart diseases; increases blood	7	Fruit	Raw fruit is consumed	Iron deficiency; Anaemia	5
*Malva neglecta* Wallr. (Malvaceae) SWAT004658	Naghan Kai^O, P^ Teekaly/Teekla^O^ De Eshaa Tala/Teekale^P^	Locally grown; wild	Roots	Decoction is made from its roots	Digestive problems; improve sexual desire	4	Roots	Decoction is made from its roots	Digestion problems; improve sexual desire	5
			Fruit	Fruit is eaten raw	Diabetes		Fruit	Fruit is eaten raw	Diabetes	
			Leaves	Leaves are consumed raw or as cooked vegetables	Urinary tract infections		Leaves	Leaves are consumed raw or as cooked vegetables	Urinary tract infections	
*Musa* × *paradisiaca* L. (Musaceae) SWAT004662	Keela^O, P^	Bought	Fruit	Fruit is consumed raw	Loose motions; vomiting	1	Fruit	Fruit is consumed raw	Loose motions; vomiting	2
*Morus nigra* L. (Moraceae) SWAT001596	Ghras tuth^O^	Locally grown; wild	Fruit	Fruit is consumed raw	Respiratory tract infections	1	–	–	–	–
*Nasturtium officinale* R.Br (Brassicaceae) SWAT004663	Tarmera^O^ Dalamera^P^	Locally grown; wild	Leaves	Leaves are consumed as a salad	Digestive problems	9	Leaves	Leaves are consumed as a salad	Digestion problems	9
*Nigella sativa* L. (Ranunculaceae) SWAT001601	Kalwanji^O, P^	Bought	Seeds	Seeds are added to vegetables or eaten with water or milk	Chest infections; COVID-19; respiratory tract infections	1	Seeds	Seeds are added to vegetables or eaten with water or milk	Chest infections	1
*Olea europaea* subsp. *Cuspidata* (Wall. &G.Don) Cif. (Oleaceae) SWAT004666	Shalwanai^O^ Shawan^P^	Locally grown; wild	Fruit	Fruit is consumed raw	Weakness; increases blood; constipation; diabetes; blood pressure	18	Fruit	Fruit is consumed raw	Weakness; increases blood; constipation; diabetes; blood pressure	13
*Oryza sativa* L. (Poaceae) SWAT004667	Rezan^O^ Varize^P^	Bought	Seeds	Seeds are boiled in water; the water is then cooled and used to wash the head	Hair loss	1	Seeds	Seeds are boiled in water; the water is then cooled and used to wash the head	Hair loss	1
*Oxalis corniculata* L. (Oxalidaceae) SWAT004668	Tuftufak^O^ Tarweekai^P^	Locally grown; wild	Leaves	Leaves are consumed as snacks	Digestive problems	1	Leaves	Fresh leaves are consumed raw	Digestion problems	2
*Ocimum basilicum* L. (Lamiaceae) SWAT004664	Bobrai^O, P^ Babari^O^	Bought	Seeds	Seeds are consumed with water	Digestive problems	1	Seeds	Seeds are consumed with water	Digestion problems; blood pressure	1
*Portulaca oleracea* L. (Portulacaceae) SWAT004665		Locally grow; wild	Leaves	Leaves are consumed with water	Digestive problems	1	Leaves	Seeds are consumed with water	Digestion problems; blood pressure	1
*Phaseolus vulgaris* L. (Fabaceae) SWAT004670	Mait^O, P^	Locally grown; cultivated	Seeds	Seeds are consumed as vegetables	Anaemia	7	Seeds	Seeds are consumed as vegetables	Anaemia	5
*Phoenix dactylifera* L. (Arecaceae) SWAT004671	Khajora^O^ Khajeera^P^	Bought	Fruit	Fresh or dried fruit is consumed, preferably with milk	General body tonic; sexual tonic; immuniser	5	Fruit	Fresh or dried fruit is consumed, preferably with milk	General body tonic; sexual tonic; immuniser	2
*Pinus gerardiana* Wall. ex D.Don (Pinaceae) SWAT004672	Zanguzai^O, P^	Locally grown; wild	Fruit	Fruit is consumed raw	General weakness; energiser	11	Fruit	Fruit is consumed raw	General weakness; energiser	8
*Pinus* spp. (Pinaceae) SWAT004673	Nashtar^O^	Locally grown; wild	Resin	Resin is dried and used with water	Fractures	1	–	–	–	–
*Piper nigrum* L. (Pinaceae) SWAT004674	Gharas Mruch^O^ Toor Mirch^P^	Bought	Seeds	Seeds are consumed with a small amount of water or consumed with vegetables	General body tonic; skin roughness; night blindness	17	Seeds	Seeds are consumed with a small amount of water or consumed with vegetables	General body tonic; skin roughness; night blindness	16
*Plantago lanceolata* L. (Plantaginaceae) SWAT004675	Aspeghul^O^	Locally grown; wild	Seeds	Seeds are consumed with water	Constipation	1	–	–	–	–
*Polygonatum verticillatum* (L.) All. (Asparagaceae) SWAT004676	Meeralam^O, P^	Locally grown; wild	Leaves	Dried leaves are consumed with water	Sexual tonic; immuniser; aphrodisiac	13		Dried leaves are consumed with water	Sexual tonic; immuniser; aphrodisiac	7
				Leaves are dried and powdered and then used topically	Skin diseases					
*Prunus dulcis* D.A.Webb (Rosaceae) SWAT004677	Badam^O^	Locally grown; cultivated	Fruit	Fruit is dried and consumed	Improve eyesight; immuniser; sexual tonic; enhance memory	1	–	–	–	–
*Prunus persica* (L.) Batsch (Rosaceae) SWAT004678	Shamtulai^O^	Locally grown; cultivated	Fruit	Fruit is consumed raw	Diabetes	1	–	–	–	–
*Psidium guajava* L. (Myrtaceae) SWAT004679	Amrod^O, P^	Bought	Fruit	Fruit is consumed raw	Constipation	7	Fruit	Fruit is consumed raw	Constipation	5
*Punica granatum* L. (Lythraceae) SWAT004680	Naskaraf^O^ Narsavai^P^	Locally grown; wild	Fruit cover	Dried fruit cover is eaten with water or mixed with yoghurt and consumed	Abdominal pain	11	Fruit cover	Dried fruit cover is eaten with water or mixed with yoghurt and consumed	Abdominal pain	9
*Peganum harmala* L. (Nitrariaceae) SWAT004669	Sponda^O^ Sapelani^P^	Locally grown; wild	Leaves	Leaves are consumed with a small amount of water	Joint pain	2	–	–	–	–
			Aerial parts	Leaves are consumed with a small amount of water	Abdominal gas					
*Quercus baloot* Griff. (Fagaceae) SWAT001589	Sat/Chatt^O^ Cheere/Serray^P^	Locally grown; wild	Fruit	Fruit is roasted	Diabetes	10	Roots	Roots are made into a decoction and consumed twice a day	Diabetes	8
			Roots	Roots are made into a decoction	Gastric problems					
*Rumex dentatus* L. (Polygonaceae) SWAT001591	Zando^O^ Zunda^P^	Locally grown; wild	Leaves	Leaves are ground and applied topically	Wound healing	2	Leaves	Leaves are ground and applied topically	Wound healing	3
				Leaves are dried and consumed with water	Pain			Leaves are dried and consumed with water	Pain	
*Ricinus communis* L. (Euphorbiaceae) SWAT001603	Baghdavan^O^	Bought	Leaves	Leaves are consumed raw	Body pain	1	–	–	–	–
*Raphanus* *raphanistrum* subsp. *sativus* (L.) Domin (Brassicaceae) SWAT001590	Molia^O^ Meele^P^	Locally grown; cultivated	Roots	Roots are consumed as a salad	Hepatitis; abdominal gas; digestive problems	7	Roots	Roots are consumed as a salad	Hepatitis; abdominal gas; digestive problems	6
*Senegalia modesta* (Wall.) P.J.H.Hurter (Fabaceae) SWAT004623	Palwasa^O^	Locally grown; wild	Resin	Dried resin eaten with water	Nightfall; sexual tonic	1	–	–	–	–
*Sageretia thea* *(*Osbeck*)* M.C.Johnst*.* (Rhamnaceae) SWAT001602	Mamorye^O^	Locally grown; wild	Seeds	Seeds are consumed with a small amount of water	Malarial fever	1	–	–	–	–
*Saccharum officinarum* L. (Poaceae) SWAT001592	Ghana^O, P^	Bought	Aerial parts	Juice is extracted and consumed	Liver problems; refrigerant	8	Aerial parts	Juice is extracted and consumed	Liver problems; refrigerant	5
*Solanum americanum* Mill. (Solanaceae) SWAT001593	Warghusti^O^	Locally grown; wild	Fruit	Fruit is consumed raw	Digestive problems	1	–	–	–	–
*Teucrium stocksianum* Boiss. (Lamiaceae) SWAT004656	Kasturai^O, P^	Locally grown; wild	Leaves	Leaves are consumed with water	Abdominal gas; dysentery; Malaria; diabetes; typhoid; blood pressure; body pain	13	Leaves	Leaves are boiled in water, made into decoction	Abdominal gas; dysentery; malaria; diabetes; typhoid; blood pressure; body pain	8
*Tamarix aphylla* (L.) H.Karst. (Tamaricaceae) SWAT001594	Ghaz^O, P^	Locally grown; wild	Aerial parts	Dried and then put in oil, applied topically on the body	Burns; wounds	1	Aerial parts	Dried and then put in oil, applied topically on the body	Burns; wounds	1
*Taraxacum campylodes* G.E.Haglund (Compositae) SWAT001595	Zer Gul^O^	Locally grown; wild	Flowers; leaves	Both leaves and flowers are consumed with water	Infected wounds	1	–	–	–	–
*Thymus linearis* Benth. (Lamiaceae) SWAT001595	Mizbuk/izbuk^O^ Marveeje^P^	Locally grown; wild	Seeds	Seeds directly consumed with water	Abdominal pain; indigestion	15	Seeds	Seeds directly consumed with water	Abdominal pain and gas	17
*Urtica dioica* L. (Urticaceae) SWAT001598	Dhur^O^ Sezinkaiy^P^	Locally grown; wild	Leaves	Fresh leaves are eaten in very low quantities with bread	Stop loose motions; indigestion	2	Leaves	Fresh leaves are eaten in very low quantities with bread	Stop loose motions; indigestion	2
				Decoction	Urinary tract infections; refrigerant			Decoction	Urinary tract infections; refrigerant	
*Vitis vinifera* L. (Vitaceae) SWAT001599	Angeer^O, P^	Bought	Fruit	Fruit is consumed raw	General body tonic; indigestion; constipation	12	Fruit	Fruit is consumed raw	General body tonic; indigestion; constipation	10
*Visnaga daucoides* Gaertn. (Apiaceae) SWAT004633	Speerkey^O^	Bought	Seeds	Seeds are consumed raw	Ear infection	1	–	–	–	–
*Vachellia nilotica* (L.) P.J.H.Hurter & Mabb. (Fabaceae) SWAT004624	Kikar^O^	Locally grown; wild	Resin	Dried resin eaten with water	Sexual tonic	1	–	–	–	–
*Withania* *coagulans* (Stocks) Dunal (Solanaceae) SWAT001597	Shapyango^O^ Shapyanga^P^	Bought	Leaves	Leaves are boiled in water and then everything is consumed	Refrigerant; abdominal gas	16	Leaves	Leaves are dried, powdered and consumed with water	Refrigerant	17
*Zingiber officinale* Roscoe (Zingiberaceae) SWAT001600	Adrak^O, P^	Bought	Tubers	Tea is made or they are used with vegetables	Chest infections	4	Tubers	Tea is made or they are used in vegetables	Chest infections	3
*Fungi*										
*Morchella esculenta* (L.) Pers. (Morchellaceae) SWAT004660	Gargicho^O^ Kargicho^P^	Wild	Fruit body	Aerial part are consumed with water	Sexual tonic; immuniser	2	Flowers	Flowers are consumed with water	Sexual tonic; immuniser	2

The Ormur community reported medicinal uses for all of the quoted plant taxa belonging to the thirty-nine families, with the dominant families being Amaryllidaceae and Lamiaceae. In contrast, the medical ethnobotany of Pathans consisted of only fifty-two species distributed across thirty-four families.

A large number of plant species were used for digestive and liver problems. We observed that *Allium sativum* (12 URs) and *Apteranthes tuberculata* (6 URs) were the most commonly used taxa to treat these ailments. Other essential disease categories included genital and cardiovascular problems, which were treated by many plant species. The prevalence of these two different kinds of diseases could be because they are common in the study area, or medicinal plants are more useful and have potent activity against digestive problems. We may assert that traditional herbal therapies may play an influential role in treating digestive tract problems, as they are the target of valuable activity [30]. Some of the most important and commonly used plants included *Withania* *coagulans* (33 URs), *Piper nigrum* (33 URs), and *Thymus linearis* (32 URs), *Olea europaea* subsp. *cuspidata* (31 URs), *Allium sativum* (26 URs), *Mentha longifolia* (26 URs), *Ficus carica* (24 URs), *Vitis vinifera* (22 URs), *Polygonatum verticillatum* (20 URs), *Punica granatum* (20 URs), *Teucrium stocksianum* (21 URs), *Pinus gerardiana* (19 URs), *Nasturtium officinale* (18 URs), *Quercus baloot* (18 URs), and *Apteranthes tuberculata* (16 URs). The large number of use reports for the taxa mentioned above indicates the cultural acceptability of these taxa across the two groups.

Sixty-eight taxa processed as herbal therapies were taken orally, while the remaining six were applied topically to treat skin diseases. Some plants were perceived as effective in treating COVID-19, which indicates how new plant uses emerged during this emergency and how this novel knowledge rapidly circulated across the communities. We have also provided the correspondence between emic disease names and etic disease categories (Table 2).

**Table 2:** Correspondence between emic disease names and etic disease categories and their frequency of mention

Digestive	Respiratory	Cardiovascular
Abdominal gas (11) Abdominal pain (12) Acidity (1) Appetiser (2) Colic pain (2) Constipation (12) Digestion problems (26) Dysentery (2) Gastric problems (3) Indigestion (5) Loose motion (1) Loss of appetite (1)Piles (2) Stop loose motion (3) Vomiting (2) Worm infestation (1)	Cough (1) Chest infection (7) COVID-19 (4) Respiratory tract infection (5) Cold fever (1) Common cold (2) Remove sputum (1)	Blood pressure (6) Heart diseases (3)
Blood	Endocrine	Musculoskeletal
Anaemia (3) Increases blood (5) Blood thinner (3)	Diabetes (20)	Join pain (4)
Skin	Female genital	Male genital
Burns (2) Face smoothness (1) Fracture (1) Hair loss (3) Infected wound (1) Pus (1) Skin diseases (1) Skin dryness (1) Skin rashes (4) Skin roughness (2) Snake bite (1) Wound (6)	Abnormal menstruation (1) Leucorrhoea (2)	Infertility (1) Improve sexual desire (5) Sexual tonic (10) Night fall (1)
Ear	Urological	Neurological
Ear infection (1)	Diuretic (2) Kidney problems (1) Nocturnal enuresis (in children) (2) Urinary tract infection (6)	Brain tonic (2) Enhance memory (1)
Eye	Liver	General
Improve eyesight (2) Night blindness (2)	Hepatitis (4) Liver problems (2)	Believed to be a strong antibiotic (1) Body pain (4) Cosmetics (1) Energiser (3) Fever (6) General body tonic (9) General weakness (3) Immune booster (5) Immuniser (6) Infected teeth (3) Iron deficiency (2) Malaria (7) Obesity (3) Pain (4) Refrigerant (9) Tonic (1) Typhoid (6) Weakness (11)

Some of the used plants were spices or condiments that were store-bought, such as *Allium sativum*, *Coriandrum sativum*, *Curcuma longa*, *Ferula assa-foetida*, *Piper nigrum*, and *Zingiber officinale*. These spices and flavouring agents have been medicinal since the Middle Ages [31]. It has been stated that the spices are both grown in the Middle East and imported from Africa and Southeast Asia. Their trade may be a legacy from Roman times, when black pepper, ginger, turmeric, and cardamom were transported from Southeast Asia into the Mediterranean via Arabian incense trade routes [32, 33]. Plant availability is a crucial factor shaping traditional plant use. Participants also quoted several fruits that are available in the local market. Some of the plants bought from the market or “*Pinsar”* shops are collected in other regions of North-West Pakistan, especially Upper Chitral and Gilgit-Baltistan.

Differences in plant availability between rural and urban contexts may also account for the differences in the plant lists reported in this study and the published literature. In the current survey, 40 plants were collected in the wild, and eight were cultivated. At the same time, 26 were bought or imported, which indicates that the knowledge of the studied communities is quite heterogeneous and likely to have been acquired from outside the region. The cross-cultural overlap among the different medicinal plants having different origins is presented in the next section.

### Cross-cultural analysis

**Fig. 3:**
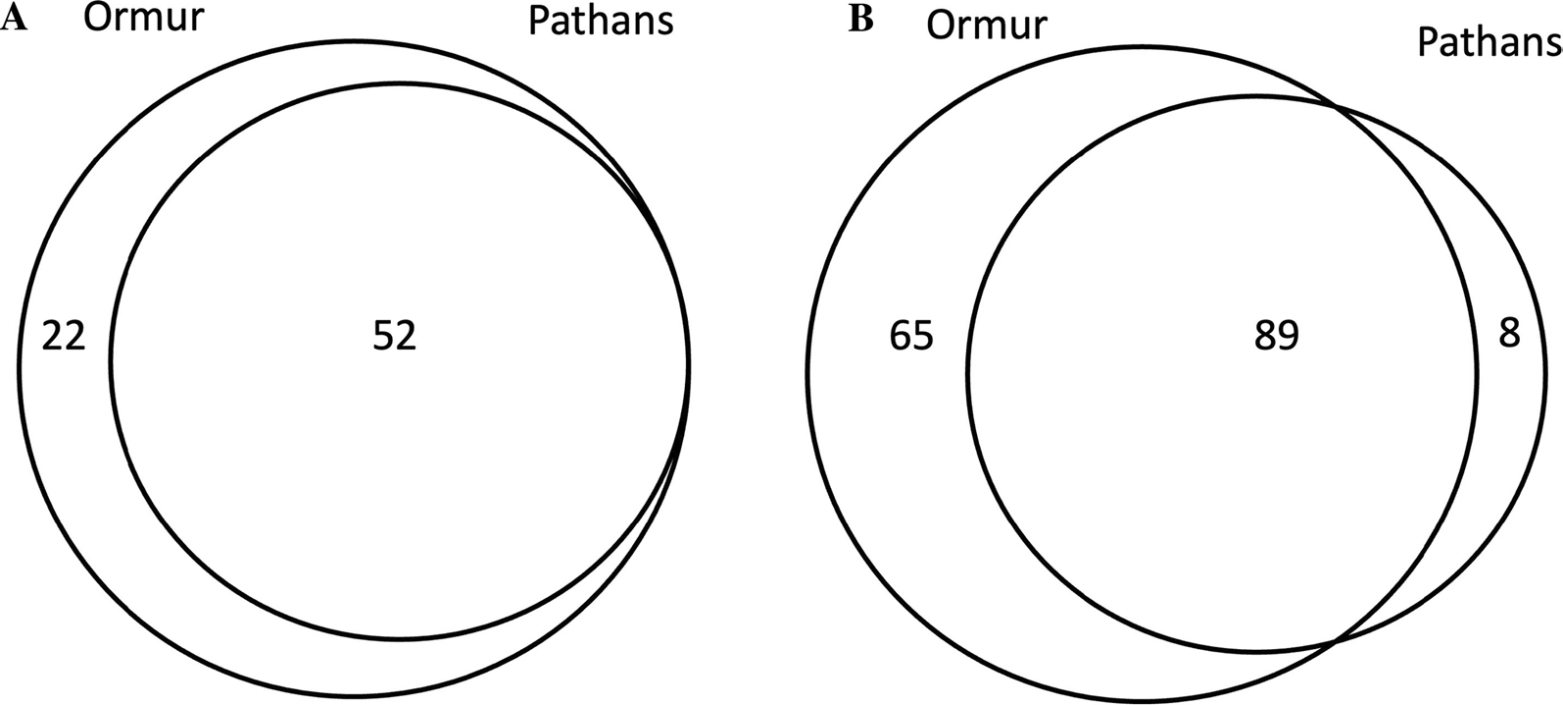
Venn diagram: **a** revealing the number of unique and common species among Ormurs and Pathans, **b** showing the number of species used in the same (shared) or different ways (not shared) between the two ethnic groups

**Fig. 4:**
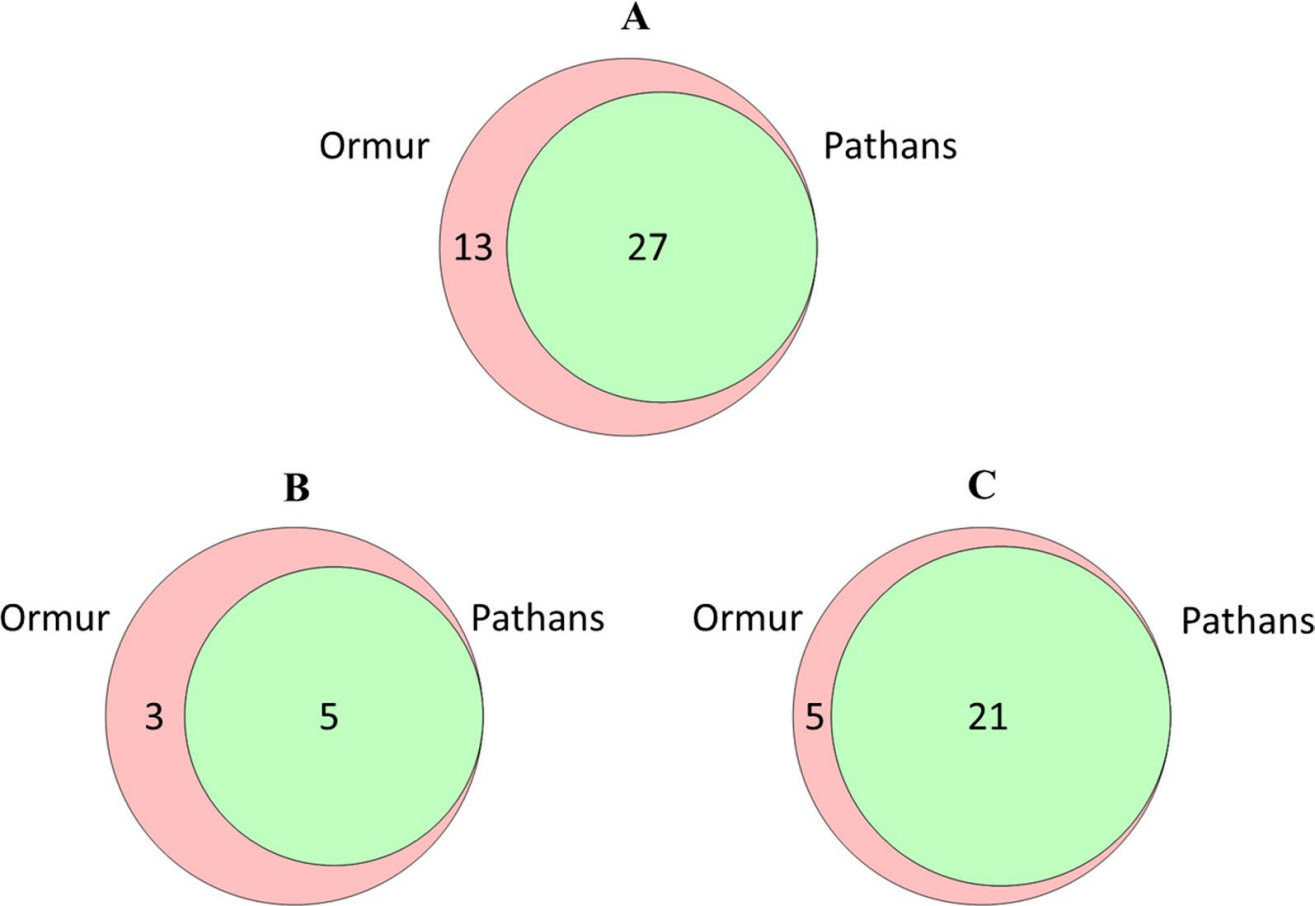
The overlap between the use of medicinal plants which were **a** collected in the wild, **b** cultivated, and **c** imported from other areas

The cross-cultural comparison revealed considerable overlap in the use of plants among the two groups (Fig. 3a); however, we observed a higher ratio of heterogeneity among the uses of commonly reported plants (Fig. 3b). Pathans exclusively quoted a few uses, while Ormurs have retained several idiosyncratic medicinal uses for the respective plants that make their contemporary medical ethnobotany different from that of Pathans (Fig. 4a). These results suggest that Ormurs may have retained a strong attachment to their original traditional medical system despite being a linguistic minority in the region. They have also learned medicinal plant knowledge from Pathans, as these groups have lived together for centuries and mutually negotiated and exchanged. This finding contradicts the widely accepted paradigm that the knowledge of linguistic minorities tends to adapt with that of the majority group in multicultural environments and retain negligible idiosyncratic uses. Here, we see a paradigm shift that could have some implications for the Ormur. For instance, the strong attachment of the Ormur community to their quoted medicinal plants may be used to inform policymakers to focus on their plant-based cultural heritage since these may also interfere with the use of and adherence to prescribed biomedicine [34].

The current research findings also show that Ormur multilingualism may have enhanced the incorporation of Pathan folk remedies into their repertoire: those plants “of their own origin” plus those of Pathans, with whom they have lived for centuries. We argue that Ormurs may have absorbed Pathan’s knowledge of medicinal plants as they can speak Pashto, and thus, they might have learned from Pathans. Pathans, however, cannot speak the Ormuri language; therefore, they do not have the same ability to access medicinal knowledge of Ormur regarding medicinal plants.

Their local plant nomenclature seems to confirm that since Ormurs have adopted the plant names of Pathans. (47 plant names are shared with Pathans.) The first author, whose mother tongue is a Waziristani dialect of Pashto, could easily recognise that Ormurs adopted plant names from Pashto as widely spoken in the entire area of Waziristan and Kaniguram. In our previous ethnobotanical study of wild food plants, our research group found several idiosyncratic uses among Ormurs [6], thus confirming the hypothesis of double ethnobotany, including original elements and Pathan/Pashto elements. Even though the Ormur retains more knowledge of medicinal plants as compared to Pathans, it is worth mentioning that local ethnobotanical knowledge of medicinal plants is highly threatened among both communities, where we could see that most of the plants were reported by only one or two informants among both ethnic groups. However, Ormurs still retain local knowledge of medicinal plants, demonstrating their strong attachment to the traditional medical system until recently. For the last two decades, large-scale mass migration has been driven by the “war on terror”, which has caused local communities to move out of these areas, ultimately affecting the daily life activities, local cultural practices, and natural resource management of Ormurs and Pathans, which in turn may have negatively impacted the traditional knowledge on natural resources of the two linguistic groups.

It is worth mentioning, however, that the characteristic uses of these plants among the Ormur may represent their ancestral knowledge and the knowledge that was possibly learned to some extent from different sources. It is also important to note that all the idiosyncratic uses of medicinal plants quoted by the Ormur people were only reported by a single informant, except two taxa, *Adiantum capillus-veneris* and *Peganum harmala,* which were cited by 4 and 2 participants, respectively. Very few of the reported plants mentioned by the participants were currently used; most were used in the past. One of the main reasons for the erosion of local plant knowledge may be the globalisation and capillary diffusion of modern medical systems, which has led people to rely on Western biomedicines. Ormurs reported more use reports (346 URs) than Pathans (261 URs), although both communities have equal access to the official health system in the study area. This difference might be due to the Ormur historical socio-cultural stratification of their LEK/TEK and their disadvantaged economic conditions, resulting in possibly less access to the modern healthcare system, making them more dependent on local medicinal plants.

We also compared the wild, cultivated, and imported plants among the two studied communities. We observed that Ormurs used more medicinal taxa in each of the three categories, thus indicating a comprehensive, rich knowledge of medicinal plants.

### Local ecological knowledge and language: a mutating complex

LEK is a mutating complex that changes over time and space. In today’s world, local plant knowledge of medicinal resources is highly vulnerable to change as modern and Western medicines have changed the global trend, and people rely more on these medicines, causing them to abandon traditional herbal therapies. For instance, during the survey, many participants confirmed that they do not need to prepare any traditional treatments at home as they may find doctors and medical practitioners in local clinics and pharmacies. People also asserted that extensive outmigration due to the “war on terror”—the guerrilla initiated by religious extremist groups two decades ago—has driven people to live in cities where they are more exposed to allopathic medicine, and this has greatly influenced medicinal plant knowledge, as evidenced by the fact that people have difficulty recalling the local names of medicinal plants. They have likely forgotten much of the local medicinal knowledge related to local plant resources, and we may expect that medicinal knowledge associated with the more common and frequently used taxa still resides in their memories, which they reported in this study.

The drivers of change in local plant knowledge might differ between the two communities. For instance, the medical ethnobotany of the two communities may have been affected by the introduction of exotic uses of the reported plants, as we observed the impact of the dominant Pathan culture on Ormurs. Therefore, not only can we say that the idiosyncratic uses of specific plants quoted by Ormurs cannot be considered cultural markers, but that they might have acquired local plant knowledge from other cultural groups through occasional intermarriage or symbiotic relationships with them, as also explained by the results of our previous studies [3, 35–37].

We observed that Ormurs have mostly adopted local Pathan names for the reported plant species, and we have recorded twenty-seven idiosyncratic plant names among Ormur (Table 3). Earlier, studying wild food ethnobotany among the same groups, we found that some phytonyms were linked to Persian and Kurdish plant names [6]. The use of Pashto as the *lingua franca* formed the foundation for sharing local knowledge on medicinal plants and has caused the homogenisation of knowledge of the quoted medicinal plants. Linguistic adaptation represents one type of socio-cultural adaptation made during socio-cultural negotiations, which can be observed in recent times via intermarriages. During our previous food ethnobotanical survey, we found that the Ormur people are more exposed to other cultural groups as they have migrated from their hometown to nearby cities because of the unstable security situation in the area, and now their knowledge and language are more threatened than ever before (see also [6]). Many participants had forgotten some important linguistic terms, others could not name some important plants, and others mentioned misleading local names for certain more popular plants, all revealing the dire situation of the minority language and related cultural knowledge. This finding also indicates linguistic homogenisation among the individuals of the two groups and the dominant impact of Pashto, the *lingua franca*, in the study area.

**Table 3:** The list of the plants that reported with different names among the two communities

Botanical name	Recorded local name
*Allium longifolium*	Khukh^O^ Yov-ree^P^
*Allium sativum*	Ozzha^O^ Yaza^P^
*Amaranthus blitum*	Sakaak^O^ Ranzaka^P^
*Anacardium occidentale*	Kajo/Badyan^O^
*Cannabis sativa*	Bangara^O^
*Cassia fistula*	Chumbarkhayan^O^ Turlargai^P^ Ghras goon^O^
*Cocos nucifera*	Kopra^O, P^ Gari^O^
*Curcuma longa*	Guleskhand^O^ Korkaman^P^
*Ficus carica*	Inzir^O^ Tugha^P^
*Ferula assa-foetida*	Eng^O^ Aang^P^
*Lepidium draba*	Ghargasti^O^
*Mentha longifolia*	Gwan^O^ Welanai^P^
*Malus pumila*	Meleez^O^ Manra^P^
*Morus nigra*	Ghras tuth^O^
*Nasturtium officinale*	Tarmera^O^ Dalamera^P^
*Olea europaea subsp. cuspidata*	Shalwanai^O^ Shawan^P^
*Oryza sativa*	Rezan^O^ Varize^P^
*Oxalis corniculata*	Tuftufak^O^ Tarweekai^P^
*Pinus gerardiana*	Zanguzai^O, P^
*Piper nigrum*	Gharas Mruch^O^ Toor Mirch^P^
*Punica granatum*	Naskaraf^O^ Narsavai^P^
*Quercus baloot*	Sat/Chatt^O^ Cheere/Serray^P^
*Rumex dentatus*	Zando^O^ Zunda^P^
*Ricinus communis*	Baghdavan^O^
*Raphanus raphanistrum subsp. sativus*	Molia^O^ Meele^P^
*Thymus linearis*	Mizbuk/izbuk^O^ Marveeje^P^
*Urtica dioica*	Dhur^O^ Sezinkaiy^P^

^O^ = Ormur; ^P^ = Pathans

Morgenstierne [7] asserted that Ormuri speakers of both Kaniguram and Logar in Afghanistan have been significantly affected by their Pashtun neighbours and have freely borrowed numerous words from them. Kieffer [38] declared that the Ormuri language has reached the final stage of its resistance; it is used only in the home, and even there, due to exogamous marriages, its use is diminishing.

Many elderly Ormur participants mentioned that their close relationships with Pathans had influenced language and local ecological knowledge. In contrast, others revealed that migration further assisted the loss of mental lexicons, including terms related to local plants and their uses. Stringer affirmed the mental lexicon as a storehouse of traditional ecological knowledge (for an in-depth discussion, see [39] and references therein). He further argues that preserving the environment is essential for revitalising languages attached knowledge. Overall, the “war on terror” in Waziristan has had a significant negative impact on the traditional plant knowledge of the local population, and much of this knowledge is at risk of being lost permanently. We observed that the post-war rehabilitation process has caused significant changes in daily life practices, which may have affected local ecological knowledge.

Even today, the security situation is highly fragile and local communities tend to live in safe areas or have migrated. We found that the younger generations do not hold traditional medicinal knowledge, and they often leave mountain areas and move to cities. Scientists have affirmed that language has a vital role in shaping local ecological knowledge, which also includes the use of medicinal plants [40–43].

The rapid mass migration from the area has become one of the main factors weakening the connection between people and nature, ultimately impacting the local plant knowledge. In the present study, we discovered that a different factor contributes to the mutation of local ecological knowledge. The study area has suffered from the “war on terror” for the last two decades. Local communities have been migrating to cities and leaving the area for years. They may have also learned knowledge from other cultural groups with whom they have interacted.

Thus, we can see that the LEK of the studied communities is threatened [6] as the citation frequency is minor for each plant.

### LEK and its conservation

The conservation of local medical knowledge in medical education, particularly that related to providing public healthcare to local communities, especially minorities and other underprivileged communities, has to consider these communities’ cultural knowledge, beliefs, and practices [44–46]. In the study area, biocultural heritage related to medicinal plants in the two communities is facing severe threats, especially among Ormurs, a linguistic minority, who are under twofold pressure. On the one hand, their medicinal knowledge is gradually fading. At the same time, they have adopted the Pashto language and have forgotten much of the local plant nomenclature (see, for instance, [6]). In this regard, conserving and revitalising cultural knowledge through possible institutions is deemed necessary, and ethnobotanists should play an important role in convincing the local educational authorities to make the LEK part of educational activities. Indeed, among ethnobotanists, the narrative of conservation through education has been gaining considerable attention. However, most of society, including botany students, still does not know the importance of local or traditional medicinal knowledge. Therefore, certain initiatives will have to be adopted not only to protect local plant knowledge from further erosion, but it will also improve the quality of primary health care through cultural competency training. Above all, policymakers must be sincere in tackling the extinction of local knowledge systems and must support and design culturally sensitive educational and medical programmes. Moreover, culturally competent healthcare fosters sensitivity to the cultural context of sickness and healing, including self-treatment with medicinal plants. It encourages practitioners to negotiate treatment acceptable to both clinician and patient [46].

Local people must take an active part in protecting biocultural heritage and natural resources. This will effectively happen if we educate people about the cultural, ecological, and economic values of natural resources and the impacts of human activities on them, as well as teach them the skills and knowledge they need to conserve resources effectively. Different platforms could be utilised; for instance, place-based courses on biocultural conservation can be taught in schools, and in an informal way, communities could also be involved through public awareness campaigns and on-the-ground conservation training for local communities. In particular, local schools are a vital platform for learning and revitalising local plant knowledge, as we have some insight from previous research work in the Hindukush [47].

According to the biological convention’s message, we must cross many obstacles to promote healthy local ecological practices. As stated by the UN Agenda 2030, it is important to preserve cultural heritage for social sustainability and to protect nature and the ecosystem [48]. The issue of conservation cannot be addressed by merely revolving around academia. Still, instead, we need bold steps to promote and disseminate botanical information in schools and other educational institutions. Several scientists have clearly articulated this and have made it clear that it has clear policy implications [18, 49, 50]. Until we educate our society about the cultural and economic importance of protecting local cultural resources and promoting the knowledge related to these natural resources, our claim to be effective in conserving nature is hollow. For a sustainable future on this planet, everyone has to be concerned in this regard. Ecological transition is a timely call for which we must take on board the local communities to make them economically independent. Brayboy and Castagno [51] stated that indigenous ways of knowing are neither inferior nor superior to Western ways; they are different perspectives that need to be acknowledged rather than trying to justify their inclusion in Western education. Therefore, we argue that if the curriculum is aligned with local cultural and ecological realities, it will promote children’s connection to science, which is crucial but missing in schools where examples are infrequent and usually used only to illustrate isolated concepts [52]. In the ongoing context, if medicinal plants and their commercialisation are valued, we can obtain very positive results in alleviating poverty, and it will also help biocultural conservation. More people will be connected to nature and the local resources, which is important for the social sustainability of this highly fragile mountain region, as the large mass migration from the area is posing serious population threats to nearby urban areas.

### Limitations of the study

In this study, we only interviewed the male community members. We were not allowed to conduct interviews with women because of the strict practice of *Parda* (“veil”), but we may expect that medicinal knowledge is quite gendered, and women could be more knowledgeable than men. Many research studies have demonstrated the important role of women in holding and retaining medicinal plant knowledge [53–60]. It has been affirmed that medicinal plant knowledge is most often the cultural domain of women because of their role in providing care within the household [53–55, 61, 62], which includes, as part of their labour and domestic activities, the management of plant-based resources, leading to extensive knowledge of locally practised herbal therapies [53, 54, 58]. Thus, they retain more knowledge of medicinal plants [53, 55–57], and their knowledge could be epistemologically different than that of men [54].

## Conclusion

The current study revealed a significant homogenisation of local medicinal knowledge among the two studied ethnic groups. Ormurs have retained comparatively rich expertise of the quoted plants compared to Pathans, which, on the one hand, might suggest that they have kept their traditional medical knowledge. On the other hand, it may indicate that they have absorbed a significant body of exotic knowledge on the recorded medicinal plants, thus proving our hypothesis that the medicinal plant knowledge of Ormur has been significantly affected by their close neighbours. However, it is interesting to note that the local plant nomenclature among Ormurs has been highly influenced by Pathans, demonstrating their socio-cultural adaptation, which might be a driver of learning exotic medicinal knowledge regarding natural resources. This study indicates that local plant knowledge is highly threatened due to the invasion of Western medicine into the area during the last few decades. Other drivers of change might include the extensive mass migration from the area over the previous two decades because of the ongoing “war on terror”, which has limited local communities’ access to traditional lands and natural resources, making medicinal plant knowledge more fragile. We suggest that policy measures should be taken to preserve the fading cultural heritage and help conserve the threatened medicinal flora across the region. Further ethnographic research should be conducted, especially studies that focus on recording the medicinal plant knowledge among women, as they are considered potential knowledge holders of herbal medicines. It is equally important to investigate the local plant knowledge in other parts of Waziristan to preserve the fading cultural heritage among Pathans, which is highly threatened.

## Data Availability

All the required data are provided in the article.

## Abbreviations

LEK: Local ecological knowledge
TEK: Traditional ecological knowledge
URs: Use reports
WFPs: Wild food plants

## References

[1] Mustafa B, Hajdari A, Pieroni A, Pulaj B, Koro X, Quave CL (2015). A cross-cultural comparison of folk plant uses among Albanians, Bosniaks, Gorani and Turks living in south Kosovo. J Ethnobiol Ethnomed.

[2] Ceuterick M, Vandebroek I, Torry B, Pieroni A (2008). Cross-cultural adaptation in urban ethnobotany: the Colombian folk pharmacopoeia in London. J Ethnopharmacol.

[3] Aziz M, Abbasi AM, Ullah Z, Pieroni A (2020). Shared but threatened: the heritage of wild food plant gathering among different linguistic and religious groups in the Ishkoman and Yasin Valleys. North Pak Foods.

[4] Aziz AM, Ullah Z, Adnan M, Sõukand R, Pieron A (2022). Plant use adaptation in Pamir: Sarikoli Foraging in the Wakhan Area. North Pak Biol.

[5] Jernigan K (2012). Plants with histories: the changing ethnobotany of iquito speakers of the peruvian amazon. Econ Bot.

[6] Aziz MA, Ullah Z, Al-Fatimi M, De Chiara M, Sõukand R, Pieroni A (2021). On the trail of an ancient Middle Eastern Ethnobotany: traditional wild food plants gathered by Ormuri speakers in Kaniguram. NW Pakistan Biol.

[7] Morgenstierne G. Parachi and Ormuri (Volume 1). In Indo-Iranian Frontier Languages; H. Aschehoug & Co, Oslo, Norway. 1929. https://archive.org/details/in.gov.ignca.14416/page/n69/mode/2up. Assessed 24 Sep 2023.

[8] Leech R (1838). A vocabulary of the baraky language. J R Asiat Soc Bengal.

[9] Campbell L, Lee NH, Okura E, Simpson S, Ueki K. 2022. The Catalogue of Endangered Languages (ElCat). http://endangeredlanguages.com/userquery/download/, Accessed 28 Aug 2022.

[10] Quave CL, Pieroni A (2015). A reservoir of ethnobotanical knowledge informs resilient food security and health strategies in the Balkans. Nat Plants.

[11] Kalle R, Sõukand R, Pieroni A (2020). Devil is in the details: Use of wild food plants in historical Võromaa and Setomaa, present-day Estonia. Foods.

[12] Hopping K, Yangzong C, Klein J (2016). Local knowledge production, transmission, and the importance of village leaders in a network of Tibetan pastoralists coping with environmental change. Ecol Soc.

[13] Mamedov N, Gardner Z, Craker LE (2005). Medicinal plants used in Russia and Central Asia for the treatment of selected skin conditions. J Herbs Spices Med Plants.

[14] Saslis-Lagoudakis CH, Hawkins JA, Greenhill SJ, Pendry CA, Watson MF, Tuladhar-Douglas W, Baral SR, Savolainen V (2014). The evolution of traditional knowledge: environment shapes medicinal plant use in Nepal. Proc R Soc B: Biol Sci.

[15] Sõukand R, Pieroni A (2016). The importance of a border: Medical, veterinary, and wild food ethnobotany of the Hutsuls living on the Romanian and Ukrainian sides of Bukovina. J Ethnopharmacol.

[16] Pieroni A, Sõukand R (2017). Are borders more important than geographical distance? The wild food ethnobotany of the Boykos and its overlap with that of the Bukovinian Hutsuls in Western Ukraine. J Ethnobiol.

[17] Sõukand R, Kalle R, Pieroni A (2022). Homogenisation of biocultural diversity: plant ethnomedicine and its diachronic change in Setomaa and Võromaa, Estonia, in the last century. Biology.

[18] Olsson P, Folke C (2001). Local ecological knowledge and institutional dynamics for ecosystem management: a study of Lake Racken watershed. Sweden Ecosyst.

[19] Weckerle CS, de Boer HJ, Puri RK, van Andel T, Bussmann RW, Leonti M (2018). Recommended standards for conducting and reporting ethnopharmacological field studies. J Ethnopharmacol.

[20] Heinrich M, Lardos A, Leonti M, Weckerle C, Willcox M, Applequist W, Stafford G (2018). Best practice in research: consensus statement on ethnopharmacological field studies–ConSEFS. J Ethnopharmacol.

[21] Khattak S (2011). Ormuri: The Silent Victim of Militancy.

[22] Grierson GA (1918). The ormuri or bargista language: an account of a little-known eranian dialect. Mem R Asiat Soc Bengal.

[23] Morgenstierne, G. Report on a Linguistic Mission to Afghanistan; Instituttet for Sammenlignende Kulturforskning: Oslo, Norway. 1926. https://archive.org/details/dli.pahar.2283. Assessed on 24 Sep 2023.

[24] Efimov, V.A. The Ormuri Language in Past and Present; Forum for Language Initiative: Islamabad. 2011. https://theswissbay.ch/pdf/Books/Linguistics/Mega%20linguistics%20pack/Indo-European/Iranian/Ormuri%20Language%20in%20Past%20and%20Present%20%28Efimov%29.pdf

[25] Kieffer CL (1979). fin proche des langues iraniennes résiduelles du Sud-Est, ṛ etč en Afghanistan. Lang Société.

[26] Kieffer CL (1972). multilinguisme des Ormuṛs de Baraki-Barak (Afghanistan). Studia Iran.

[27] Farooq S, Barki A, Khan MY, Fazal H (2012). Ethnobotanical studies of the flora of tehsil Birmal in South Waziristan Agency. Pakistan Pak J Weed Sci Res.

[28] International Society of Ethnobiology. ISE Code of Ethics (with 2008 Additions). Available online: http://ethnobiology.net/code-of-ethics (Accessed on 10 June 2023).

[29] Flora Online database [24] (http://www.worldfloraonline.org).

[30] Cárdenas PA, Kratz JM, Hernández A, Costa G, Ospina LF, Baena Y, Simões CMO, Jimenez-Kairuz Á, Aragon M (2017). In vitro intestinal permeability studies, pharmacokinetics and tissue distribution of 6-methylcoumarin after oral and intraperitoneal administration in Wistar rats. Braz J Pharm Sci.

[31] Freedman P (2015). Health, wellness and the allure of spices in the middle ages. J Ethnopharmacol.

[32] Groom N (1981). Frankincense and myrrh: a study of the Arabian incense trade.

[33] Van der Veen M, Morales J (2015). The Roman and Islamic spice trade: new archeological evidence. J Ethnopharmacol.

[34] Vandebroek I, Balick MJ (2012). Globalisation and loss of plant knowledge: challenging the paradigm. PLoS ONE.

[35] Aziz MA, Ullah Z, Pieroni A (2020). Wild food plant gathering among kalasha, yidgha, nuristani and khowar speakers in chitral. NW Pakistan Sustainability.

[36] Kalle R, Sõukand R (2016). Current and remembered past uses of wild food plants in Saaremaa, Estonia: changes in the context of unlearning debt. Econ Bot.

[37] Pieroni A, Sõukand R (2019). Ethnic and religious affiliations affect traditional wild plant foraging in Central Azerbaijan. Genet Resour Crop Evol.

[38] Kieffer C (1977). The approaching end of the relict southeast Iranian languages Ormuri and Paraci. Int J Sociol Lang.

[39] Stringer D When Grasshopper Means Lightning: How Ecological Knowledge is Encoded in Endangered Languages. 2016. https://terralingua.org/langscape_articles/when-grasshopper-means-lightning-how-ecological-knowledge-is-encoded-in endangered-languages/ (Accessed on 24 Sep 2023).

[40] Maffi L (2005). Linguistic, cultural and biological diversity. Annu Rev Anthropol.

[41] Menendez-Baceta G, Aceituno-Mata L, Reyes-García V, Tardío J, Salpeteur M, Pardo-de-Santayana M (2015). The importance of cultural factors in the distribution of medicinal plant knowledge: a case study in four Basque regions. J Ethnopharmacol.

[42] Pieroni A, Quave CL (2005). Traditional pharmacopoeias and medicines among Albanians and Italians in Southern Italy: a comparison. J Ethnopharmacol.

[43] Pieroni A, Sõukand R, Bussmann RW (2020). The inextricable link between food and linguistic diversity: wild food plants among diverse minorities in Northeast Georgia. Caucasus Econ Bot.

[44] Balick MJ, Kronenberg F, Ososki AL, Reiff M, Fugh-Berman A (2000). Medicinal plants used by Latino healers for women's health conditions in New York City. Econ Bot.

[45] Pieroni A, Vandebroek I (2009). Traveling Cultures and Plants: The Ethnobiology and Ethnopharmacy of Human Migrations.

[46] Juckett G (2005). Cross-cultural medicine. Am Fam Phys.

[47] Aziz MA, Volpato G, Fontefrancesco MF (2022). Perceptions and revitalization of local ecological knowledge in four schools in Yasin Valley, North Pakistan. Mt Res Dev.

[48] United Nations. Transforming Our World: The 2030 Agenda for Sustainable Development. Resolution adopted by the General Assembly on 25 September 2015, A/RES/70/1. New York, NY: United Nations. 2015.https://sdgs.un.org/2030agenda. Accessed on 24 Sep 2023.

[49] Berkes F, Jolly D (2001). Adapting to climate change: social-ecological resilience in a Canadian western Arctic community. Conserv Ecol.

[50] Perales H, Benz BF, Brush SB (2005). Maise diversity and ethnolinguistic diversity in Chiapas, Mexico. Proc Natl Acad Sci USA.

[51] Brayboy BMJ, Castagno AE (2008). Indigenous knowledge and native science as partners: a rejoinder. Cult Stud Sci Educ.

[52] Aikenhead GS, Roth W-M, Désautels J (2002). Whose scientific knowledge? The coloniser and the colonised. Science Education as/for Sociopolitical Action.

[53] Voeks RA (2007). Are women reservoirs of traditional plant knowledge? Gender, ethnobotany and globalisation in northeast Brazil. Singap J Trop Geogr.

[54] Howard P. The major importance of "minor" resources: women and plant biodiversity. London: International Institute for Environment and Development. 2003. http://www.jstor.org/stable/resrep01807. Accessed 24 Sep 2023.

[55] Coe FG, Anderson GJ (1996). Ethnobotany of the Garífuna of eastern Nicaragua. Econ Bot.

[56] Gollin L. The taste and smell of Taban Kenyah (Kenyah medicine): an exploration of chemosensory selection criteria for medicinal plants among the Kenyah Leppo'Ke of East Kalimantan, Borneo, Indonesia. PhD dissertation. Ann Arbor: Bell & Howell; 1997.

[57] Kainer KA, Duryea ML (1992). Tapping women's knowledge: plant resource use in extractive reserves. Acre Brazil Econ Bot.

[58] Razafindraibe M, Kuhlman AR, Rabarison H, Rakotoarimanana V, Rajeriarison C, Rakotoarivelo N, Randrianarivony T (2013). Medicinal plants used by women from Agnalazaha littoral forest (South-eastern Madagascar). J Ethnobiol Ethnomed.

[59] Begossi A, Hanazaki N, Tamashiro JY (2002). Medicinal plants in the Atlantic Forest (Brazil): knowledge, use, and conservation. Hum Ecol.

[60] Qureshi RA, Ghufran MA, Gilani SA, Yousaf Z, Abbas G, Batool A (2009). Indigenous medicinal plants used by local women in southern Himalayan regions of Pakistan. Pak J Bot.

[61] Teixidor-Toneu I, Martin GJ, Puri RK, Ouhammou A, Hawkins JA (2017). Treating infants with frigg: linking disease aetiologies, medicinal plant use and care-seeking behaviour in southern Morocco. J Ethnobiol Ethnomed.

[62] Wayland C (2001). Gendering local knowledge: medicinal plant use and primary health care in the Amazon. Med Anthropol Q.

